# Allele frequencies in the *VRN*-*A1*, *VRN*-*B1* and *VRN*-*D1* vernalization response and *PPD*-*B1* and *PPD*-*D1* photoperiod sensitivity genes, and their effects on heading in a diverse set of wheat cultivars (*Triticum aestivum* L.)

**DOI:** 10.1007/s11032-014-0034-2

**Published:** 2014-02-05

**Authors:** Tibor Kiss, Krisztina Balla, Ottó Veisz, László Láng, Zoltán Bedő, Simon Griffiths, Peter Isaac, Ildikó Karsai

**Affiliations:** 1 Centre for Agricultural Research, Agricultural Institute, Hungarian Academy of Sciences, Martonvásár, 2462 Hungary; 2 John Innes Centre, Norwich Research Park, Norwich, NR4 7UH UK; 3 IDna Genetics Ltd, Norwich Research Park, Norwich, NR4 7UH UK

**Keywords:** Vernalization genes, Photoperiod response, DEV49–DEV59 plant development phases, Wheat (*T. aestivum* L.)

## Abstract

Heading of cereals is determined by complex genetic and environmental factors in which genes responsible for vernalization and photoperiod sensitivity play a decisive role. Our aim was to use diagnostic molecular markers to determine the main allele types in *VRN*-*A1*, *VRN*-*B1*, *VRN*-*D1*, *PPD*-*B1* and *PPD*-*D1* in a worldwide wheat collection of 683 genotypes and to investigate the effect of these alleles on heading in the field. The dominant *VRN*-*A1*, *VRN*-*B1* and *VRN*-*D1* alleles were present at a low frequency. The *PPD*-*D1a* photoperiod-insensitive allele was carried by 57 % of the cultivars and was most frequent in Asian and European cultivars. The *PPD*-*B1* photoperiod-insensitive allele was carried by 22 % of the genotypes from Asia, America and Europe. Nine versions of the *PPD*-*B1*-insensitive allele were identified based on gene copy number and intercopy structure. The allele compositions in *PPD*-*D1*, *PPD*-*B1* and *VRN*-*D1* significantly influenced heading and together explained 37.5 % of the phenotypic variance. The role of gene model increased to 39.1 % when *PPD*-*B1* intercopy structure was taken into account instead of overall *PPD*-*B1* type (sensitive vs. insensitive). As a single component, *PPD*-*D1* had the most important role (28.0 % of the phenotypic variance), followed by *PPD*-*B1* (12.3 % for *PPD*-*B1*_overall, and 15.1 % for *PPD*-*B1*_intercopy) and *VRN*-*D1* (2.2 %). Significant gene interactions were identified between the marker alleles within *PPD*-*B1* and between *VRN*-*D1* and the two *PPD1* genes. The earliest heading genotypes were those with the photoperiod-insensitive allele in *PPD*-*D1* and *PPD*-*B1*, and with the spring allele for *VRN*-*D1* and the winter alleles for *VRN*-*A1* and *VRN*-*B1*. This combination could only be detected in genotypes from Southern Europe and Asia. Late-heading genotypes had the sensitivity alleles for both *PPD1* genes, regardless of the allelic composition of the *VRN1* genes. There was a 10-day difference in heading between the earliest and latest groups under field conditions.

**Electronic supplementary material:**

The online version of this article (doi:10.1007/s11032-014-0034-2) contains supplementary material, which is available to authorized users.

## Introduction

Bread wheat is grown approximately between latitudes 60°N and 40°S in the temperate zone (Nuttonson 1955). These regions exhibit considerable differences in macro- and microclimate, requiring a wide range of genetic diversity if the crops are to be capable of acclimatization. Detailed knowledge of the physiological and genetic factors influencing the start and length of the flowering period could contribute to the successful breeding of genotypes better able to adapt to present and future changes in the environment.

The molecular basis of the complex genetic regulation of the flowering period has largely been clarified in wheat and barley (Cockram et al. 2007; Distelfeld et al. 2009), but there is still much uncertainty about the adaptation of these species to a wide range of environmental factors and about the molecular and genetic processes taking place in the plants due to interactions between these factors. One of the most important components of adaptation is flowering time, which is determined to a great extent by gene groups that regulate the vernalization requirement, i.e. the cold period that induces the transition from the vegetative to the generative phase (*VRN* genes), and the photoperiod sensitivity (*PPD* genes) (Worland 1996; Dubcovsky et al. 1998; Worland et al. 1998). In the case of wheat, several gene families are involved in the genetic regulation of the vernalization response. Those with the greatest effect are *VRN*-*A1*, *VRN*-*B1* and *VRN*-*D1*, which are located on the long arm of the homologous chromosomes 5A, 5B and 5D (Pugsley 1971; Law et al. 1975; Galiba et al. 1995; Worland 1996; Barrett et al. 2002; Yan et al. 2003). Depending on the ratio of dominant and recessive alleles in the *VRN* genes in the three genomes of hexaploid wheat, it is possible to distinguish cultivars with winter (recessive) or spring (dominant) seasonal growth habit, while genotypes with the facultative habit have various combinations of dominant and recessive alleles. Numerous polymorphisms have been found in the promoter, exon and intron regions of the *VRN*-*A1* gene, which include duplications and deletions. The basic allele types of spring/winter habit are associated with various sequence differences detected in the promoter region and with relatively large insertions or deletions in the intron1 region (Yan et al. 2004; Fu et al. 2005), based on which several haplotypes have been identified. Thus, the promoter region of the *VRN*-*A1a* allele is duplicated, while the *VRN*-*A1b* allele differs from the *vrn*-*A1* (recessive) allele in a 20-bp deletion in the TC-repetitive elements of the 5′ untranslated region. The alleles *vrn*-*A1c* (a 7,222-bp deletion in the intron1 region), *Vrn*-*A1d* (a 32-bp deletion in the promoter region) and *Vrn*-*A1e* (a 54-bp deletion in the promoter region) have been described in tetraploid wheat (Yan et al. 2004; Fu et al. 2005). The *VRN*-*A1a* and *VRN*-*A1b* alleles have been found to be associated with the dominant *VRN*-*A1* haplotypes, and the *vrn*-*A1c* allele with the recessive haplotype (Sherman et al. 2004). To date, the correlation between the *VRN*-*A1d* and *VRN*-*A1e* alleles and spring (dominant) habit has not been proven experimentally (Yan et al. 2004; Fu et al. 2005). A much smaller extent of polymorphism has been demonstrated for the *VRN*-*B1* and *VRN*-*D1* genes, and for these two genes the spring/winter type can basically be attributed to an insertion/deletion in the intron1 region (Fu et al. 2005; Milec et al. 2013). The dominant *VRN*-*A1a* allele has the most pronounced genetic effect in the development of spring habit. Plants bearing this allele require no cold treatment at all to flower. By contrast, the dominant *VRN*-*B1* and *VRN*-*D1* genes only partially eliminate the need for cold treatment before the generative phase begins (Pugsley 1971, 1972; Kato et al. 2001; Loukoianov et al. 2005).

In wheat the most important genes regulating photoperiod sensitivity are *PPD*-*A1*, *PPD*-*B1* and *PPD*-*D1*, which are located on the homologous chromosomes 2A, 2B and 2D, respectively (Law et al. 1978; Börner et al. 1993). Based on the distribution of the alleles of these genes, temperate zone cereals can be divided into photoperiod-sensitive and -insensitive groups. The heading of genotypes carrying the photoperiod-insensitive allele is rapid regardless of whether they are exposed to short-day or long-day illumination. The presence of the photoperiod-sensitive allele, however, substantially delays heading in the case of short days. From the point of view of functional polymorphism, the least information is available for the *PPD*-*A1* gene. Only a few polymorphisms have been detected up to now, but none of these have been strictly linked to functionality (Beales et al. 2007). In durum wheat (*T. durum* Desf.), on the other hand, photoperiod sensitivity has been attributed to a mutation in the *PPD*-*A1* gene. The two deletions described in this case (1,027 and 1,117 bp) are located in the same region as the large loss of sequences noted for the *PPD*-*D1a* allele (Wilhelm et al. 2009). More information is available on the functional polymorphisms in *PPD*-*B1* on the 2B chromosome. Díaz et al. (2012) described a point mutation in the exon3 region in cv. Chinese Spring, and proved that a higher copy number of the *PRR* (pseudo-response regulator) gene is responsible for photoperiod insensitivity. These mutations were observed to co-segregate with the early heading phenotype (Beales et al. 2007; Díaz et al. 2012; Nishida et al. 2013). Beales et al. (2007) detected a deletion measuring 2,089 bp in the promoter region of the *PPD*-*D1a* gene, but only in the photoperiod-insensitive allele variant of the gene. This deletion was found to induce a change in the molecular mechanism regulating gene expression, the phenotypic manifestation of which was early heading under both short- and long-day illumination. Several other polymorphisms have also been identified that differentiate various photoperiod-sensitive haplotype in *PPD*-*D1* (Guo et al. 2010). It is generally accepted that the most intense genetic effect is exerted by the dominant *PPD*-*D1a* allele, followed by the dominant *PPD*-*B1a* and *PPD*-*A1a* alleles (Blake et al. 2009; Díaz et al. 2012).

The latest models for the genetic control of flowering in cereals suggest a functional relationship between the *PPD1* and *VRN1* genes (Cockram et al. 2007; Distelfeld et al. 2009). It has already been reported that in barley the various allele combinations of these genes result in diverse plant development categories (Karsai et al. 2008) and that under controlled environmental parameters the effects of the *VRN* and *PPD* genes exhibit a significant difference in agronomic traits that are definitely determined by the flowering date (Laurie et al. 1994; Karsai et al. 1999, 2006; Snape et al. 2001). Under field conditions, however, the various environmental factor combinations experienced in different years result in considerable variability in the phenotypic effects of the individual alleles of these genes, often leading to contradictory findings (Snape et al. 1985; Worland 1996; Worland et al. 1998; Kato et al. 2000). In addition, although a lot of information is available on the allele compositions and effects of individual vernalization response and photoperiod sensitivity genes separately, there are much less data available on the frequency distributions and phenotypic effects of the various allele combinations in the three *VRN*-*1* and in the two *PPD*-*1* genes together (Blake et al. 2009; Andeden et al. 2011; Iqbal et al. 2011; Díaz et al. 2012).

The aim of the study reported here, carried out on a wide range of wheat varieties, was (1) to use molecular markers to characterize the major alleles of genes responsible for vernalization response and photoperiod sensitivity; (2) to investigate the territorial distribution of various allele groups over the five continents, in five geographical regions of Europe; (3) to evaluate data on field-grown plants to determine possible correlations between the alleles and the time required for heading.

## Materials and methods

All plant samples (521 from Europe, 62 from Asia, 6 from Africa, 90 from America, 4 from Australia) originated from the winter wheat gene bank of the Agricultural Institute (MTA ATK, Martonvásár, Hungary) and were chosen on the basis of breeding location, pedigree and flowering data recorded in previous experiments. The genotypes included both old and new breeding materials, of which some used to be grown widely and some are important in current wheat production systems. The aim was to include a heterogeneous gene pool in the experiment that could be expected to include all the main allele types of the genes in question (Table 1).

**Table 1:** Geographic distribution of the dominant alleles of the vernalization response (*VRN*) and photoperiod sensitivity (*PPD*) genes

Continent	Ratio of genotypes	Number of examined samples from each continent	*VRN*-*A1* spring allele	*VRN*-*B1* spring allele	*VRN*-*D1* spring allele	*PPD*-*B1a* insensitive allele	*PPD*-*D1a* insensitive allele
Europe	76 %	521	2 % (12^a^)	3 % (16)	3 % (15)	17 % (88)	58 % (301)
Asia	9 %	62	10 % (6)	11 % (7)	24 % (15)	61 % (38)	79 % (49)
Africa	1 %	6	50 % (3)	33 % (2)	0 %	17 % (1)	17 % (1)
America	13 %	90	16 % (14)	20 % (18)	9 % (8)	26 % (23)	37 % (33)
Australia	1 %	4	75 % (3)	50 % (2)	0 %	25 % (1)	75 % (3)
Number and ratio of each column	683	6 % (38)	7 % (45)	6 % (38)	22 % (151)	57 % (387)	

^a^Number in parenthesis is the number of genotypes

**Fig. 1:**
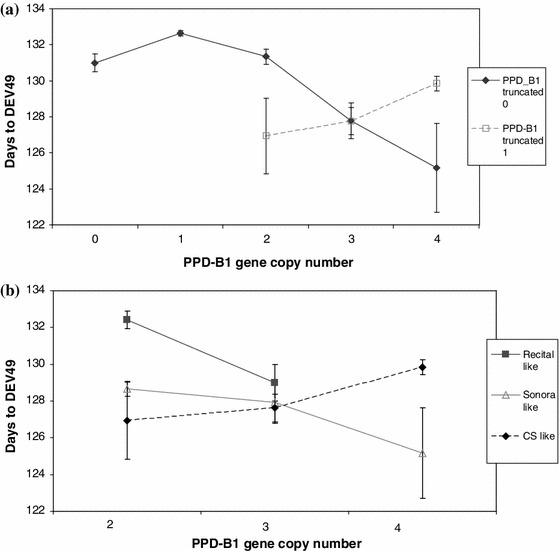
Effect of haplotype combinations in *PPD*-*B1* on DEV49 (phase at which spike is located in the upper part of the flag-leaf sheath; 49 = number of days required from 1 January to reach this stage) based on the factorial GLM analysis of variance (ANOVA) in the group of 683 wheat accessions. Associations between the presence/absence of the truncated gene and gene copy number (**a**) and between intercopy structure and gene copy number (**b**). *PPD* photoperiod sensitivity genes, *VRN* vernalization response genes

Genomic DNA was extracted from young leaves (100 mg) using the DNeasy^®^ Plant Mini kit (Qiagen, Hilden, Germany) according to the manufacturer’s instructions. The alleles were determined using gene-specific molecular markers based on published results. Three primer pairs were employed to detect the allele variants of the *VRN*-*A1* gene. All genotypes were analysed with the primer pair VRN1AF and VRN1-INTR1R, which is linked to the promoter region and allows the dominant *VRN*-*A1a*, *VRN*-*A1b*, *VRN*-*A1c* and recessive *vrn*-*A1* alleles to be detected (Yan et al. 2004; Fu et al. 2005). Two other primer pairs were used to distinguish between the *VRN*-*A1c* and *vrn*-*A1* alleles. The first primer pair was Intr1/A/F2 and Intr1/A/R3, which is linked to the intron1 region and is suitable for the demonstration of the *VRN*-*A1c* allele, and the second primer pair was Intr1/C/F and Intr1/AB/R, which detects the lack of the intron1 deletion and was used as the positive control (Yan et al. 2004; Fu et al. 2005). The allele variants of the *VRN*-*B1* gene were tested with two primer pairs: Intr1/B/F and Intr1/B/R3 is linked to the intron1 region and enables the dominant *VRN*-*B1* allele to be detected, while Intr1/B/F and Intr1/B/R4 was used as the positive control to confirm the absence of the deletion (Yan et al. 2004; Fu et al. 2005). Two primer pairs were also applied to distinguish the allele variants of the *VRN*-*D1* gene. One consisted of Intr1/D/F and Intr1/D/R3, which is linked to the intron1 region and detects the dominant *VRN*-*D1* allele, while the primer pair Intr1/D/F and Intr1/D/R4 was taken as the positive control. The allele variants of the *PPD*-*D1* gene were detected with the primers of Ppd-D1F, Ppd-D1R1 and Ppd-D1R2 (Beales et al. 2007). Various combinations of these primers allowed a large deletion in the promoter of the *PPD*-*D1* gene to be detected, with which the photoperiod-insensitive and sensitive alleles could be distinguished (Beales et al. 2007; Faure et al. 2007; Yang et al. 2009). The copy number of the *PPD*-*B1* gene was estimated relative to the reference gene TaCO2 using a multiplex TaqMan^®^ assay as described by Díaz et al. (2012). For those genotypes having more than one copy of the *PPD*-*B1* gene, further PCR assays were carried out to characterize their intercopy types. Ppd-B1exon3SNP forward and reverse primers were used as a control to prove the presence–absence of the *PPD*-*B1* gene (Beales et al. 2007). Three additional primer pairs were also used: (1) Ppd-B1_2ndcopy_F1 and Ppd-B1_2ndcopy_R1, characterizing the intercopy region of the truncated and intact copies of the cv. Chinese Spring allele (Beales et al. 2007; Díaz et al. 2012); (2) PpdB1_F25-R70, characterizing the intercopy region between the intact gene copies of the cv. Chinese Spring allele; (3) PpdB1_F31-R36, characterizing the Sonora/Timstein type of intercopy region (Díaz et al. 2012) [Electronic Supplementary Material (ESM) Fig. 1]. PCR amplification was performed on GeneAmp^®^PCR System 9700 instruments (Applied Biosystems, Foster City, CA). The PCR protocols used for the individual primer pairs were based on information from the literature. The products were separated on 1.0 or 2.0 % agarose gels (depending on the expected fragment size). The gel images were visualized and photographed with G:BOX iChemi (Syngene, Synoptics Group, Cambridge, UK) UV equipment. The molecular data for genotypes available in international databases were used as controls in the molecular marker-assisted analysis of the alleles. The classification of geographical regions in Europe was based on the reports of Feekes (1978), Roussel et al. (2005) and Balfourier et al. (2007).

The heading date of each genotype was determined in autumn-sown wheat in field experiments at the Agricultural Institute (MTA ATK), Martonvásár (Central Hungary) in 2011 and 2012. The sowing dates were 16 October 2011 and 6 October 2012. Scoring was carried out in two developmental phases: DEV49 (spike located in the upper part of the flag-leaf sheath) and DEV59 (spike fully emerged from the flag leaf sheath; based on Tottman and Makepeace 1979). The DEV49 and DEV59 phases were determined in terms of the number of days required from 1 January to reach the given stage of development. The experimental plots measured 1 × 2 m, and two rows were sown for each genotype, with a between-row distance of 20 cm.

For statistical analyses, the data matrices of gene alleles were transformed into one-digit decimal numbers. For the vernalization response loci, 1 stood for winter alleles and 2 for spring alleles; for the basic photoperiod, type 1 stood for a sensitive allele and type 2 for an insensitive allele. In the case of *PPD*-*B1*, the analyses included the basic separation of sensitive–insensitive alleles and the further separation of insensitive alleles by the presence–absence of a truncated gene copy, copy number and intercopy structure type. The geographic origin was also digitalized, with the following codes: 1, West-Europe; 2, Central-Europe; 3, East-Europe; 4, South-Europe; 5, Southeast-Europe; 6, America; 7, Asia; 8, Africa; 9, Australia. For studying the source of variance and the associations between the different variables within the complete data matrix of the 683 wheat accessions, the principal component analysis (PCA) module of the Statistica 6 software package (StatSoft Inc., Tulsa, OK) was applied. The single gene effects on plant development were analysed via the main effects analysis of the General Linear Model (GLM), while the gene interactions were analysed via the Factorial analysis of variance (ANOVA) of the GLM of Statistica 6.

## Results

### Frequency of the *VRN1* and *PPD1* alleles in the wheat cultivar collection

Analysis performed with gene-specific molecular markers revealed the presence of the dominant (spring) *VRN*-*A1* allele in 6 % of the genotypes in our wheat genotype collection (38 genotypes), the dominant (spring) *VRN*-*B1* allele in 7 % (45) and the dominant (spring) *VRN*-*D1* allele in 6 % (38) (Table 1). Of the 38 genotypes carrying the dominant *VRN*-*A1* allele, 33 gave the product characteristic of the *VRN*-*A1a* allele, while five gave a molecular fragment characteristic of the *VRN*-*A1b* allele. No genotypes containing the dominant *VRN*-*A1c* allele were identified in the collection. The semi-dominant *PPD*-*D1a* photoperiod-insensitive allele was present in 387 genotypes (57 %), while the *PPD*-*B1a* photoperiod-insensitive allele was carried by 151 (22 %) genotypes. The *PPD*-*B1* locus proved to be quite variable. In addition to the two genotypes found to have null copy of the *PPD*-*B1* gene, nine versions of the insensitive *PPD*-*B1a* allele were identified based on copy number, the presence of a truncated copy and the junction structure between two gene copies (Table 2). With only an exception of one genotype, these genotypes represented copy number variations of two to four of the three basic types published by Díaz et al. (2012). Of the genotypes with the insensitive allele, 50.3 % were characterized by copy number variation of the cv. Chinese Spring basic allele type, 25.8 % by copy number variation of the cv. Recital basic allele type and 23.2 % by variations of the cv. Sonora/Timstein basic allele type.

**Table 2:** Variations in the *PPD*-*B1a* insensitive allele, their frequencies and geographic distributions

*Ppd*-*B1a* allele type^a^	Intercopy structure between^b^		Haploid copy number^c^	No. of genotypes	Geographic distribution			
	Truncated and intact genes	Two intact genes			America	Europe	Asia	Other
Recital	**0**	**0**	**2**	**37**	**5**	**29**	**2**	**1**
	0	0	3	2	1	–	1	–
Sonora/Timstein	0	223	2	15	12	2	1	–
	**0**	**223**	**3**	**17**	**5**	**3**	**8**	**1**
	0	223	4	3	–	1	2	–
New type	**0**	**994**	**3**	**1**	–	–	**1**	–
Chinese spring	425	994	2	6	–	2	4	–
	425	994	3	36	–	18	18	–
	**425**	**994**	**4**	**34**	–	**33**	**1**	–
Total	151	23	88	38	2			

^a^Allele nomenclature is based on Díaz et al. (2012) with the original types marked in bold

^b^Intercopy type is characterized by the fragment sizes in base pairs originating from the respective PCR diagnostic assays

^c^Evaluated using the multiplex TaqMan^®^ assay as described by Díaz et al. (2012)

In the whole wheat genotype sample there was no or only a weak correlation between the allele types of the various genes; the highest correlation value (−0.19) was between *PPD*-*B1* and *PPD*-*D1*, meaning that genotypes with the insensitive allele in *PPD*-*D1* tended to have more gene copies in *PPD*-*B1*. The data matrix of the geographic origin, the vernalization response and photoperiod sensitivity gene alleles of 683 wheat accessions were further subjected to multi-factorial analysis. The first four factors in the PCA analysis showed an Eigen value of more than or close to 1, and collectively they explained 80.6 % of the total variance (ESM Table 1). Of the active variables, the first factor, which explained 41.6 % of the total variance, showed the strongest correlation with the *PPD*-*B1* allele structure, with *r* values of between −0.87 and −0.99 (ESM Fig. 2). Factor 2 (17.6 % of total variance) correlated with *VRN*-*B1* and *VRN*-*A1* and with geographic origin in a decreasing order of magnitude. *VRN*-*D1* showed the strongest correlation with Factor 3 (*r* = −0.78), while *PPD*-*D1* showed the strongest correlation with Factor 4 (*r* = −0.76). With the exceptions of *PPD*-*B1*, all the other variables significantly correlated with more than one PCA factor in a unique pattern; thus they were definitely distinguishable from each other.

Based on the patterns of factor–variable correlations, geographic origin showed the strongest association with *VRN*-*B1* allele type, followed by *VRN*-*A1* and *VRN*-*D1* in a decreasing order. The dominant allele types of the three *VRN1* genes were found least frequently (2, 3 and 3 %, respectively) in the European samples, while they were all present at a proportion of >9 % in the other continents (Table 1). If the two continents represented by a low number of samples (Africa and Australia) were omitted from the analysis, the dominant *VRN*-*A1* and *VRN*-*B1* alleles were found more frequently in the American cultivars, while the presence of the dominant *VRN*-*D1* allele was more characteristic of the Asian genotypes. Among the major genes responsible for photoperiod sensitivity, the insensitive semi-dominant *PPD*-*D1a* allele was detected most frequently in Asian genotypes, in more than two-thirds of the cultivars, and least often in the American cultivars. The *PPD*-*B1a*-insensitive allele was found in 61 % of the Asian genotypes, followed frequencies of 26 and 17 % in the American and European genotypes, respectively. In the American genotypes, only the cv. Recital and Sonora/Timstein versions were found, while the European, and especially the Asian genotypes, were more diverse (Table 2). In the European genotypes the frequencies of the original cv. Chinese Spring and Recital haplotypes were the highest, while in the Asian genotypes a three-copy version of the cv. Chinese Spring haplotype occurred most frequently.

Based on the two basic allele types of each of the three *VRN* and two *PPD* genes, a total of 32 allele combinations could be expected to occur. Of these, only 24 were observed in the 683 cultivars examined in the study, with great differences in frequency (ESM Table 2). As the majority of the wheat genotypes in the collection had the winter habit, the three most frequent allele combinations involved the recessive alleles of all three *VRN1* genes. The two most commonly occurring groups carried the recessive (winter) allele for all three *VRN1* genes, combined with the sensitive allele type of the *PPD*-*B1* gene, while they differed in *PPD*-*D1*. Together these two groups contained 461 genotypes, representing 67 % of the whole collection. Twelve groups had a frequency of <0.5 %. Among the allele combination groups with a frequency of >0.5 %, the two rarest involved the photoperiod-sensitive alleles of the *PPD*-*B1* gene, with opposing allele compositions for the three *VRN1* loci and for the *PPD*-*D1* gene. These two groups contained a total of ten genotypes (1.5 %).

The frequency distribution of the allele groups differed not only between the continents, but also between the geographical regions of Europe (ESM Fig. 3). All 12 allele combination groups included in the analysis were present in the European genotypes, with the three largest groups being group 1 (*vrn*-*A1*, *vrn*-*B1*, *vrn*-*D1*, *ppd*-*B1*, *PPD*-*D1*), 2 (*vrn*-*A1*, *vrn*-*B1*, *vrn*-*D1*, *ppd*-*B1*, *ppd*-*D1*) and 3 (*vrn*-*A1*, *vrn*-*B1*, *vrn*-*D1*, *PPD*-*B1*, *PPD*-*D1*), with relative frequencies of 44, 33 and 11 %, respectively. In Asia, in addition to groups 1 and 3 (19 and 35 %, respectively), group 7 (*vrn*-*A1*, *vrn*-*B1*, *VRN*-*D1*, *PPD*-*B1*, *PPD*-*D1*) was also more frequent (18 %) than the other groups. On the American continent, groups 1 and 2 (18 and 28 %, respectively) and group 4 (*vrn*-*A1*, *vrn*-*B1*, *vrn*-*D1*, *PPD*-*B1*, *ppd*-*D1*) (13 %) were the most characteristic.

The allele frequency pattern of the geographical regions of Europe revealed that in the Western and Central European regions groups 1 and 2 were the most frequent (25 and 47 %, and 43 and 38 %, respectively), while in Eastern Europe, group 1 was by far the most frequent (80 %). The allele combination patterns of Eastern and South-eastern Europe were very similar, but differed from each other in the ratios of the individual groups. Groups 1, 2 and 3 were found in both regions, but while the groups were detected in ratios of 80, 7 and 12 % in Eastern Europe, these ratios were 52, 5 and 40 % in the South-eastern Europe. In Southern Europe, two allele groups stood out from the others, groups 1 and 7 (frequency of 38 and 15 %, respectively). Among the five regions of Europe, allele group 7 was only present in the breeding material of Southern Europe.

### Effects of the allele compositions of the *VRN1* and *PPD1* genes on wheat heading

In the PCA analysis the developmental phases in the two consecutive years showed a strong overlap (ESM Fig. 2; ESM Table 1), and highly significant correlations were apparent between the results of the 2 years for both developmental phases (ESM Fig. 4). In the case of DEV49, the *r* value was 0.73 and 0.70 for the DEV49 and DEV59 phases, respectively. Thus, the values averaged over the 2 years were used for studying the overall phenotypic effects of the gene alleles (ESM Table 3). In the main effects ANOVA, the winter or spring allele types of the *VRN*-*A1* and *VRN*-*B1* genes had no significant effect on heading, while the allele type in *VRN*-*D1*, *PPD*-*B1* and *PPD*-*D1* significantly influenced both developmental phases. As a single factor, *PPD*-*D1* had the largest effect on DEV49 and DEV59, explaining 25.7 and 28.0 % of the phenotypic variance, respectively. In all cases the photoperiod-insensitive allele accelerated development by an average of 3.7 days for DEV49 and 4.3 days for DEV59 (Table 3). The insensitive versus sensitive allele type of the *PPD*-*B1* gene had the second strongest effect, explaining 14.2 and 12.3 % of the phenotypic variation in DEV49 and DEV59, respectively. For this gene also, the insensitive allele accelerated development by 3.2 days for DEV49 and 3.4 days for DEV59. The effect of *VRN*-*D1* was much smaller; the phenotypic contributions of this gene were 3.6 and 2.2 %, respectively. The spring allele accelerated development by 3 days for DEV49 and 2.6 days for DEV59. Together the *PPD*-*D1*, *PPD*-*B1* and *VRN*-*D1* genes accounted for 37.9 and 37.5 % of the variance of the two phenophases.

**Table 3:** Main effects of the allele types in *VRN1* and *PPD1* genes on reaching two plant developmental phases DEV49^a^ and DEV59^a^ averaged over 2 consecutive years in the wheat germplasm collection of 683 genotypes

Genes	Level of factor	*N*	DEV49		DEV59	
			Mean	95 % confidence interval	Mean	95 % confidence interval
*VRN*-*A1*	Winter	645	131.9	131.6–132.1	140.2	139.9–140.5
	Spring	38	132.6	131.5–133.7	141.0	139.7–142.3
*VRN*-*B1*	Winter	637	131.9	131.6–132.2	140.2	139.9–140.5
	Spring	46	131.9	130.8–133.1	140.5	139.2–141.7
*VRN*-*D1*	Winter	645	132.1	131.9–132.3	140.4	140.1–140.7
	Spring	38	129.1	127.4–130.8	137.8	136.1–139.5
*PPD*-*B1*_overall	Sensitive	530	132.6	132.4–132.9	141.0	140.7–141.3
	Insensitive	153	129.4	128.8–130.0	137.6	137.0–138.3
*PPD*-*B1*_truncated^b^	0	607	132.3	132.1–132.6	140.6	140.3–141.0
	1	76	128.6	127.7–129.5	136.8	136.0–137.6
*PPD*-*B1*_copy number^c^	0	2	131.0	124.6–137.4	139.3	129.7–148.8
	1	530	132.6	132.4–132.9	141.0	140.7–141.3
	2	58	130.9	129.9–131.8	139.0	138.0–140.0
	3	56	127.8	126.6–129.0	136.3	135.2–137.4
	4	37	129.5	128.5–130.4	137.5	136.6–138.3
*PPD*-*B1*_intercopy^d^	0	532	132.6	132.3–132.9	141.0	140.7–141.3
	1	39	132.2	131.3–133.2	140.5	139.5–141.6
	2	35	128.0	126.7–129.3	136.3	134.9–137.6
	3	77	128.5	127.7–129.4	136.8	136.0–137.5
*PPD*-*D1*	Sensitive	296	134.0	133.6–134.3	142.6	142.2–143.0
	Insensitive	387	130.3	130.0–130.6	138.3	138.1–138.7

^a^DEV49 (phase at which spike is located in the upper part of the flag-leaf sheath) and DEV59 (phase at which spike is fully emerged from the flag leaf sheath; based on Tottman and Makepeace 1979). The DEV49 and DEV59 phases were determined in terms of the number of days required from 1 January to reach the given stage of development

^b^For *PPD*-*B1*_truncated: 0 and 1 stand for the absence versus presence of a truncated gene, respectively

^c^For *PPD*-*B1*_copy number: 0, 1, 2, 3, 4 stand for the actual copy number (truncated and intact) of the gene

^d^For *PPD*-*B1*_intercopy: 0 = 1 copy, 1 = Recital type intercopy, 2 = Sonora/Timstein type intercopy, 3 = Chinese Spring type intercopy, irrespective of copy number

Nine versions of the insensitive allele were identified in the *PPD*-*B1* gene, representing the copy number variations of the three original insensitive allele types described by Díaz et al. (2012). Thus, statistical analyses were also carried out to decide whether there are phenotypic differences between these allele versions. In the main effects ANOVA, *PPD*-*B1*_overall was replaced by three distinct variables: *PPD*-*B1* truncated copy, *PPD*-*B1* copy number variations and *PPD*-*B1* intercopy structure type. Despite the fact that in the single marker regression all three proved to be significant components of the two plant developmental phases, only the *PPD*-*B1* intercopy type was identified by the main effect ANOVA as a significant factor of both developmental phases (ESM Table 3), explaining 17.4 and 15.1 % of the phenotypic variance in DEV49 and DEV59, respectively. As a result of including *PPD*-*B1*_intercopy instead of *PPD*-*B1*_overall into the ANOVA, the role of gene model was strengthened to a smaller extent, which now explained 40.1 and 39.1 % of the phenotypic variance in DEV49 and DEV59, respectively.

In evaluating the main factor effects of the various *PPD*-*B1* variables we found that in the case of the intercopy structure as a main effect, the cv. Recital type was significantly the latest together with the sensitivity allele, while the cvs. Sonora and Chinese Spring type were the earliest at each developmental stage (Table 3). With respect to copy numbers as main effect, the genotype with the sensitive allele (1 copy) was the latest to head, followed by the averages of two- and four-copy genotypes, while the average of genotypes with three copies of the gene was the earliest. In the case of DEV49, these values were all significantly different from each other, while in the case of DEV59 the average value of the four-copy genotypes were intermediary between those of the two- and three-copy genotypes.

Significant gene interactions were also identified with the application of factorial ANOVA; these were mostly evident between the marker alleles within the *PPD*-*B1* gene and between the *VRN*-*D1* gene and the two *PPD1* genes (ESM Table 3). In the case of *PPD*-*B1*, the gene copy number showed specific associations both with the presence–absence of the truncated gene and with the intercopy structure type (Fig. 1). In the absence of the truncated gene the increase in copy number resulted in a gradual decrease in number of days to DEV49, while the presence of the truncated gene copy resulted in a parallel increase in number of days to heading and in gene copy number. Thus, the presence of the truncated gene led to a significantly earlier DEV49 when two copies were present, but to a significantly later DEV49 when four copies were present. This opposing tendency of the cv. Chinese Spring type was also characteristic when it was compared to the intercopy type of cvs. Recital and Sonora (Fig. 1). Both for the cvs. Recital and the Sonora type structures, increase in copy number resulted in decreasing plant developmental values that significantly contrast to the values of cv. Chinese Spring types. These two opposing tendencies then led to the overall smallest values of the three copies.

**Fig. 2:**
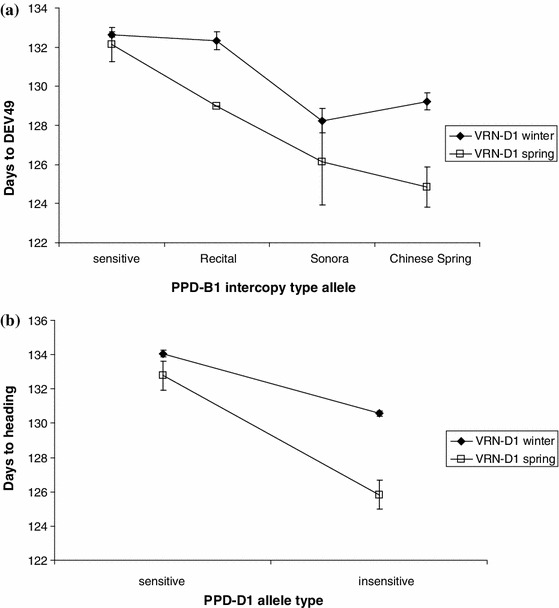
Effects of allele interactions between *VRN*-*D1* and the two photoperiod sensitivity genes on DEV49: **a** *PPD*-*B1* intercopy structure, **b** *PPD*-*D1*

In the case of *VRN*-*D1*, the insensitive alleles of both *PPD1* genes had a much stronger effect on plant development compared to the winter allele, as shown by the DEV49 values when the spring seasonal growth habit allele was present (Fig. 2). For *PPD*-*B1*, the difference between the earliest and latest alleles was 4.1 days in the genetic background of the winter *VRN*-*D1* allele, while it was 7.3 days in the background of the spring *VRN*-*D1* allele. For *PPD*-*D1*, these values were 3.5 and 7.0 days, respectively.

Averaged over the two main haplotypes in each plant, developmental genes for the two phenophases, groups 5 and 7 of the 12 main allele groups, had the earliest heading in both years (ESM Fig. 5). The mean values observed for DEV49 were 127 and 124 days for groups 5 and 7, respectively, while in the case of DEV59 these values were 136 and 133 days, respectively. The genotypes of group 5 carried the semi-dominant (photoperiod-insensitive) allele of the *PPD*-*D1* gene and the sensitive allele of the *PPD*-*B1* gene, combined with the dominant (spring) allele of the *VRN*-*D1* gene and the recessive (winter) alleles of the *VRN*-*A1* and *VRN*-*B1* genes. The genotypes in group 7 differed from those in group 5 in that the former also carried the photoperiod-insensitive allele at the *PPD*-*B1* gene. The late heading genotypes, on the other hand, had the sensitive alleles for both genes controlling photoperiod sensitivity, irrespective of the allele composition of the *VRN1* genes (groups 2, 6, 9, 10 and 12). The mean values of DEV49 and DEV59 for these groups were 134.5, and 143 days, respectively.

## Discussion

Based on the heading date characteristics of the 683 wheat accessions in the present sample, the majority of genotypes exhibited the winter seasonal growth habit, with only limited numbers of facultative genotypes (data not shown). Accordingly, analyses with gene-specific molecular markers confirmed the overwhelming presence of the winter alleles in the three *VRN1* genes, while the various dominant alleles of the *VRN1* genes were only characteristic of 6–7 % of the samples. This ratio is in good agreement with results published for winter wheat genotype collections; the allele phases in the *VRN1* genes also showed the strongest associations with the geographic origins of the genotypes (Zhang et al. 2008; Yang et al. 2009; Andeden et al. 2011). The ratio and distribution of the photoperiod-insensitive allele of the *PPD*-*D1* gene was also similar to published results (Worland et al. 1998; Seki et al. 2011; Andeden et al. 2011; Cane et al. 2013; Wilhelm et al. 2013). The photoperiod-insensitive allele of the *PPD*-*D1* gene was more frequent in the eastern, southern and south-eastern regions of Europe, while in Western Europe the photoperiod-sensitive allele of this gene has been found to be more common (Worland et al. 1998). In Central Europe the photoperiod insensitive and sensitive alleles of the *PPD*-*D1* gene occurred at similar frequencies. In the case of the *PPD*-*B1* gene, there is much less information on the occurrence, type and distribution of the insensitive allele in wheat cultivars of various geographic origins. Cane et al. (2013) studied the allele compositions of a large sample of mostly spring and facultative Australian wheat genotypes and found that 57 % of these carried various copy number variations of the insensitive allele in *PPD*-*B1*. In our study, we proved that the frequency of the photoperiod-insensitive allele was relatively high even in a mostly winter wheat germplasm. This allele was detected in 22 % of our genotype collection, in breeding materials from Asia, America and Europe, in decreasing order of frequencies. The incidence of this allele type in Europe was noted almost exclusively for genotypes from the central and south-eastern regions.

While in the cases of the *PPD*-*D1* and *PPD*-*A1*, the genetic bases of the insensitive allele could be associated with a larger deletion in the promoter region, resulting in one distinct insensitivity allele at both genes (Beales et al. 2007; Wilhelm et al. 2009; Nishida et al. 2013), for *PPD*-*B1* increases in gene copy number results in increased gene expression and, consequently, this photoperiod insensitivity (Díaz et al. 2012). This region proved to be quite variable in terms of both copy number of the gene and the type of the junctions between the various copies, probably due to the unequal crossing over (Díaz et al. 2012). In the large set of Australian spring and facultative wheats, the most frequently occurring copy number variation was three, which was characteristic of >50 % of the genotypes with the insensitive *PPD*-*B1* allele (Cane et al. 2013). This was followed by the presence of two copies, while the ratio of the insensitive genotypes with four copies was <10 %. In this winter wheat collection, the ratios of two and three copy versions of the insensitive allele were similar (around 1/3 of all the insensitive genotypes, respectively), while the ratio of the four-copy version was much higher and characteristic of about 25 % of the insensitive genotypes. The vast majority of the four-copy *PPD*-*B1* genotypes were of European origin. In both sample collections, zero copy genotypes were also identified at a very low rate (Cane et al. 2013). In this winter wheat sample, however, not only the copy number variation was characterized, but also the presence of the truncated gene and the intercopy structure type, a distinction which was not analyzed in the Australian wheat sample (Cane et al. 2013). From the aspect of intercopy structure, >50 % of the *PPD*-*B1* insensitive alleles were of cv. Chinese Spring type, while the frequencies of the cvs. Recital (European cultivar) and Sonora (Central American cultivar) types were similar to each other (26.0 vs. 23.3 %, respectively) As a result of the copy number and intercopy type, nine various versions of the insensitive allele were identified, in addition to the two genotypes with null alleles.

The results of our study of the phenotypic effects of gene allele composition proved that the allele phases in *PPD*-*D1*, *PPD*-*B1* and *VRN*-*D1* played significant roles in determining heading under field conditions. The significant phenotypic effects of *PPD*-*D1* on heading are well documented in different genetic backgrounds (Scarth and Law 1984; Law 1987; Worland 1996; Worland et al. 1998; Stelmakh 1998; González et al. 2005; Seki et al. 2011; Díaz et al. 2012). Under the environmental conditions around Martonvásár, Hungary, *PPD*-*D1* also had the largest genotypic effect on plant development under field-grown conditions, and the presence of the insensitive allele resulted in accelerated plant development in both years. These data are in line with those published by Worland et al. (1998). However, despite the *PPD*-*D1* allele type making the largest contribution to phenotypic variance in our study, its overall effect was only a 3- to 4-day difference in Central European genotypes in both years, compared to the 7.8-day difference identified between these two alleles in the INRA 372 worldwide wheat core collection studied under field conditions in UK (Wilhelm et al. 2013).

Much less is known of the phenotypic effect of the various insensitive allele types of *PPD*-*B1*, especially under field conditions (Díaz et al. 2012; Cane et al. 2013). Here we report the significant associations between the *PPD*-*B1* allele type and heading in field experiments carried out over 2 years on a large number of wheat cultivars with broad genetic diversity. In these experiments we found that the second largest phenotypic effect was due to the allele types in the *PPD*-*B1* locus and included not only the copy number but also the intercopy structure type and their interactions. Under field conditions, the intercopy structure type showed stronger associations with heading than did copy number. In general, the average values of wheat genotypes with the cv. Recital intercopy type-insensitive allele was always the latest to head, while the cv. Sonora type was the earliest. Our results, achieved in a large set of wheat genotypes, are in good agreement with those obtained in near-isogenic lines grown under short-day conditions (Díaz et al. 2012), with only difference being that in the field experiments of the wider wheat genotype set, the effect of the cv. Chinese Spring type was closer to that of the cv. Sonora type, being significantly earlier than the Recital type in most cases. It is also interesting to note that the copy number variation also resulted in altered phenotypic reaction, In general, the presence of three copies of insensitive allele resulted in the earliest heading, followed by the four-copy type; the two-copy type was the latest to head. Similar effects of copy number were identified in the Australian wheat samples (Cane et al. 2013). Our first results, however, indicated that the effect of copy number was strongly dependent on the intercopy type, a finding requiring further systematic research.

Under Central European conditions, the vernalization requirement of the winter wheat genotypes is completely saturated during the winter. Although the allele types of *VRN*-*A1*, and to a lesser extent *VRN*-*B1*, play an important role in the development of the vernalization requirement, the results obtained in our study indicate that they had no significant effect on the phenophases of DEV49 and DEV59, probably due to the saturated vernalization requirement. The spring (dominant) allele of *VRN*-*D1*, however, had a significant influence on the timing of these two phenophases, both as a main independent variable and in its significant associations with the *PPD1* genes. These findings are in line with several published results (Kato et al. 2001; Eagles et al. 2010; Cane et al. 2013). Eagles et al. (2010) proved that the spring alleles of the vernalization genes responded differently to the accumulation of vernalizing temperatures, with the common spring allele of *VRN*-*A1* showing the least response and the spring allele of *VRN*-*D1* showing a response that was similar to, but less than, a winter genotype. Thus, the dominant allele of *VRN*-*D1* was found to result in early heading in recombinant inbred lines both in a controlled environment and in autumn-sown field experiments (Kato et al. 2001) and in a larger set of Australian wheat genotypes (Eagles et al. 2010; Cane et al. 2013). Our findings on the significant associations between *VRN*-*D1* and the two photoperiod sensitivity genes are also in complete agreement with those published for the Australian wheat sample (Eagles et al. 2010; Cane et al. 2013). The effects of the insensitivity alleles of both *PPD*-*B1* and *PPD*-*D1* were significantly intensified—almost doubled—when the spring allele was present in *VRN*-*D1*, underlining the epistatic interaction between these genes.

In the case of the Australian wheat sample, the gene model based on the main allele types in the three *VRN1* and *PPD*-*D1* genes explained 45 % of the variance in heading date (Eagles et al. 2010), while the inclusion of *PPD*-*B1* copy number and a more precise resolution of the sensitive alleles of *PPD*-*D1*, together with the *VRN1* series, increased the proportion of the combined variance to 53 % (Cane et al. 2013). Here the gene model based on the main allele types of the three *VRN1* and the two *PPD1* genes explained a smaller portion of the phenotypic variance, which amounted to a maximum of 37.9 %. Also, the more precise resolution of the insensitive allele in *PPD*-*B1* increased this value only by a smaller extent, reaching 40.1 %. One of the reasons for this difference may lay in the differences between the two wheat sample collections in the ratio of genotypes with three winter alleles (true winter types) in the *VRN1* genes. This value was around 4 % in the Australian sample, while it was 85 % in the wheat sample of the present study.

As could be predicted from the gene-based model, the members of group 7 of the 12 main allele groups headed the earliest. The genotypes in this group carried the photoperiod-insensitive alleles of both the *PPD*-*D1* and *PPD*-*B1* genes, the dominant (spring) allele of the *VRN*-*D1* gene and the recessive (winter) alleles of the *VRN*-*A1* and *VRN*-*B1* genes. In the genotype collection analysed here, this group could only be detected in breeding materials from the southern region of Europe. On the other hand, late-heading genotypes were found to carry the sensitivity allele for both photoperiod sensitivity genes, irrespective of the allele combinations in the *VRN1* genes.

## Electronic supplementary material

Below is the link to the electronic supplementary material.

Supplementary material 1 (DOC 8027 kb)
