# Supplementary material for: Allele frequencies in the VRN-A1, VRN-B1 and VRN-D1 vernalization response and PPD-B1 and PPD-D1 photoperiod sensitivity genes, and their effects on heading in a diverse set of wheat cultivars (Triticum aestivum L.)
Source: Mol Breed. 2014 Feb 5;34(2):297–310. doi: 10.1007/s11032-014-0034-2 (PMC4092236; doi:10.1007/s11032-014-0034-2)
Supplement: Supplementary file 1 — Supplementary material 1 (DOC 8027 kb) [file 11032_2014_34_MOESM1_ESM.doc]

**Supplemental Materials**

**Allele frequencies in the *VRN-A1, VRN-B1* and *VRN-D1* vernalization response and *PPD-B1* and *PPD-D1* photoperiod sensitivity genes, and their effects on heading in a diverse set of wheat cultivars (*Triticum aestivum* L.)**

Tibor Kiss^1,*^, Krisztina Balla^1^, Ottó Veisz^1^, László Láng^1^, Zoltán Bedő^1^, Simon Griffiths^2^, Peter Isaac^3^, Ildikó Karsai^1^

^1^Agricultural Institute, Centre for Agricultural Research, Hungarian Academy of Sciences, H-2462 Martonvásár, Hungary

^2^John Innes Centre, Norwich Research Park, Norwich, NR4 7UH, United Kingdom

^3^IDna Genetics Ltd., Norwich Research Park, Norwich, NR4 7UH, United Kingdom

*Corresponding author: T. Kiss, E-mail: [kiss.tibor@agrar.mta.hu](mailto:kiss.tibor@agrar.mta.hu)

Supplemental **Table 1** Information on the first 6 factors of the principal component analysis (PCA) of the vernalization response and photoperiod sensitivity gene alleles and the geographic origin as active variables and the two developmental phases in two years as supplemental variables in the group of 683 wheat cultivars.

|  | **Factor 1** | **Factor 2** | **Factor 3** | **Factor 4** | **Factor 5** | **Factor 6** |
| --- | --- | --- | --- | --- | --- | --- |
| **Eigen value** | 3.7 | 1.6 | 1.0 | 0.9 | 0.7 | 0.6 |
| **Cumulative variance (%)** | 41.6 | 59.2 | 70.1 | 80.6 | 88.1 | 95.3 |
| **Factor – variable correlations** | | | | | | |
| **Origin** | -0.37 | **0.59** | ns | -0.37 | ns | **-0.61** |
| ***VRN-A1*** | ns | **0.68** | 0.45 | ns | -0.53 | 0.23 |
| ***VRN-B1*** | ns | **0.75** | ns | ns | 0.57 | 0.31 |
| ***VRN-D1*** | -0.22 | 0.31 | **-0.78** | -0.39 | ns | 0.22 |
| ***PPD-B1*_overall** | **-0.91** | ns | ns | ns | ns | ns |
| ***PPD-B1* _ truncated** | **-0.87** | ns | ns | ns | ns | ns |
| ***PPD-B1*_copy number** | **-0.96** | ns | ns | ns | ns | ns |
| ***PPD-B1*_intercopy** | **-0.99** | ns | ns | ns | ns | ns |
| ***PPD-D1*** | -0.28 | -0.39 | 0.39 | **-0.76** | ns | 0.24 |
| **DEV49_2011** | **0.48** | ns | ns | -0.42 | ns | ns |
| **DEV59_2011** | **0.42** | ns | ns | -0.42 | ns | ns |
| **DEV49_2012** | 0.38 | ns | ns | **-0.44** | ns | ns |
| **DEV59_2012** | 0.39 | ns | ns | **-0.45** | ns | ns |

Supplemental **Table 2** Predicted groups of functional allele combinations for the major *VRN1* and *PPD1* genes and those identified in the present work.

| **Group Number** | **Alleles of *VRN-A1* gene** | **Alleles of *VRN-B1* gene** | **Alleles of *VRN-D1* gene** | **Alleles of *PPD-B1* gene** | **Alleles of *PPD-D1* gene** | **Number of genotypes** |
| --- | --- | --- | --- | --- | --- | --- |
| **1 (1)** | winter | winter | winter | sensitive | insensitive | 260 |
| **2 (2)** | winter | winter | winter | sensitive | sensitive | 201 |
| **3 (3)** | winter | winter | winter | insensitive | insensitive | 86 |
| **4 (4)** | winter | winter | winter | insensitive | sensitive | 33 |
| **5 (5)** | winter | winter | spring | sensitive | insensitive | 4 |
| **6 (6)** | winter | winter | spring | sensitive | sensitive | 12 |
| **7 (7)** | winter | winter | spring | insensitive | insensitive | 13 |
| **8** | winter | winter | spring | insensitive | sensitive | 2 |
| **9** | winter | spring | winter | sensitive | insensitive | 3 |
| **10 (10)** | winter | spring | winter | sensitive | sensitive | 14 |
| **11** | winter | spring | winter | insensitive | insensitive | 3 |
| **12 (11)** | winter | spring | winter | insensitive | sensitive | 7 |
| **13 (8)** | spring | winter | winter | sensitive | insensitive | 10 |
| **14 (9)** | spring | winter | winter | sensitive | sensitive | 12 |
| **15** | spring | winter | winter | insensitive | insensitive |  |
| **16** | spring | winter | winter | insensitive | sensitive | 2 |
| **17** | winter | spring | spring | sensitive | insensitive | 1 |
| **18** | winter | spring | spring | sensitive | sensitive | 3 |
| **19** | winter | spring | spring | insensitive | insensitive | 1 |
| **20** | winter | spring | spring | insensitive | sensitive |  |
| **21** | spring | spring | winter | sensitive | insensitive | 3 |
| **22 (12)** | spring | spring | winter | sensitive | sensitive | 6 |
| **23** | spring | spring | winter | insensitive | insensitive | 1 |
| **24** | spring | spring | winter | insensitive | sensitive | 2 |
| **25** | spring | winter | spring | sensitive | insensitive |  |
| **26** | spring | winter | spring | sensitive | sensitive |  |
| **27** | spring | winter | spring | insensitive | insensitive |  |
| **28** | spring | winter | spring | insensitive | sensitive |  |
| **29** | spring | spring | spring | sensitive | insensitive |  |
| **30** | spring | spring | spring | sensitive | sensitive | 1 |
| **31** | spring | spring | spring | insensitive | insensitive | 1 |
| **32** | spring | spring | spring | insensitive | sensitive |  |

Dominant alleles

Allele combination groups identified. Numbers in brackets indicate the 12 groups analysed

Supplemental **Table 3** Analyses of variance in the developmental phases DEV49 (ear in the upper part of the flag leaf sheath) and DEV59 (ear fully emerged) averaged over two years (a) with the developmental genes as main effects and (b) with the developmental genes in factorial combinations.

| **Factors** | **DF** | **DEV49** | | **DEV59** | |
| --- | --- | --- | --- | --- | --- |
|  |  | **MS** | **P** | **MS** | **P** |
| **(a) Main effect GLM ANOVA** | | | | | |
| Intercept | 1 | 1,190,945 | 0.00000 | 1,352,257 | 0.00000 |
| *VRN-A1* | 1 | 0 | 0.867 | 0 | 0.871 |
| *VRN-B1* | 1 | 11 | 0.252 | 8 | 0.339 |
| ***VRN-D1*** | 1 | 209 | 0.00000 | 151 | 0.00009 |
| ***PPD-B1_*overall** | 1 | 699 | 0.00000 | 714 | 0.00000 |
| ***PPD-D1*** | 1 | 1898 | 0.00000 | 2530 | 0.00000 |
| Error | 677 | 8 |  | 9.6 |  |
|  |  |  |  |  |  |
| *VRN-A1* | 1 | 0.3 | 0.857 | 0.3 | 0.871 |
| *VRN-B1* | 1 | 10.9 | 0.237 | 8.8 | 0.339 |
| ***VRN-D1*** | 1 | 146.9 | 0.00002 | 115.2 | 0.00057 |
| *PPD-B1*_truncated | 1 | 8.6 | 0.295 | 1.1 | 0.733 |
| *PPD-B1*_copy number | 3 | 11.9 | 0.205 | 8.5 | 0.451 |
| ***PPD-B1*_intercopy** | 2 | 79.7 | 0.00004 | 84.6 | 0.00017 |
| ***PPD-D1*** | 1 | 1686 | 0.00000 | 2308.3 | 0.00000 |
| Error | 671 | 7.8 |  | 9.6 |  |
| **(b) Factorial effect GLM ANOVA** | | | | | |
| VRN-A1 x VRN-B1 | 1 | 16 | 0.268 | 1 | 0.759 |
| VRN-A1 x VRN-D1 | 1 | 26.6 | 0.144 | 10.3 | 0.412 |
| VRN-A1 x PPD-B1_truncated | 1 | 16.6 | 0.231 | 3 | 0.649 |
| VRN-A1 x PPD-B1_copy number | 2 | 7.5 | 0.500 | 5.4 | 0.669 |
| VRN-A1 x PPD-B1_intercopy | 3 | 5.9 | 0.641 | 1.4 | 0.958 |
| VRN-A1 x PPD-D1 | 1 | 1 | 0.820 | 1 | 0.827 |
| VRN-B1 x VRN-D1 | 1 | 44 | 0.059 | 30 | 0.158 |
| VRN-B1 x PPD-B1_truncated | 1 | 0.7 | 0.806 | 3.8 | 0.606 |
| VRN-B1 x PPD-B1_copy number | 2 | 19.9 | 0.156 | 16.4 | 0.296 |
| **VRN-B1 x PPD-B1_intercopy** | 3 | 40 | 0.0096 | 45 | 0.0154 |
| VRN-B1 x PPD-D1 | 1 | 6 | 0.416 | 2 | 0.713 |
| **VRN-D1 x PPD-B1_truncated** | 1 | 48 | 0.0397 | 57 | 0.0435 |
| VRN-D1 x PPD-B1_copy number | 3 | 291 | 0.0410 | 29.9 | 0.081 |
| **VRN-D1 x PPD-B1_intercopy** | 3 | 34.8 | 0.0172 | 33 | 0.0435 |
| **VRN-D1 x PPD-D1** | 1 | 106 | 0.0006 | 64 | 0.0153 |
| **PPD-B1_truncated x PPD-B1_copy number** | 2 | 79.6 | 0.0006 | 52.7 | 0.0196 |
| PPD-B1_truncated x PPD-B1_intercopy* | 0 |  |  |  |  |
| PPD-B1_truncated x PPD-D1 | 1 | 27 | 0.086 | 51 | 0.0289 |
| PPD-B1_copy number x PPD-B1_intercopy | 3 | 29.4 | 0.037 | 27.4 | 0.0981 |
| PPD-B1_copy number x PPD-D1 | 4 | 11.9 | 0.218 | 15.6 | 0.182 |
| PPD-B1_intercopy x PPD-D1 | 3 | 11 | 0.241 | 23 | 0.093 |
| Error | 677 | 8 – 13 |  | 10 – 16 |  |

* analysis could not be carried out due to too many missing subgroups

Supplemental **Fig. 1** Polymerase chain reaction amplification using primer pairs (A) Ppd-B1exon3SNP_F and Ppd-B1exon3SNP_R1, (B) PpdB1_F25 and PpdB1_R70, (C) PpdB1_F31 and PpdB1_R36 to detect the different allele types at the *Ppd-B1* locus.


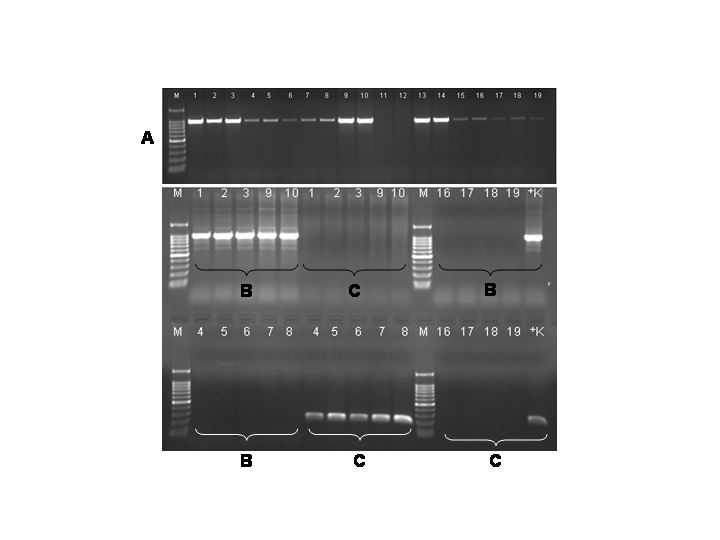


Legend: M: DNA ladder (100 bp); ^+^K: positive control; 11 and 12 represent the 0 copy type of *Ppd-B1* gene; samples of 1; 2; 3; 9 and 10 represent the ‘Chinese Spring’ allele type, samples of 4; 5; 6; 7 and 8 represent the ‘Sonora64’/‘Timstein’ allele type, samples of 16 to 19 indicate the ‘Récital’ allele type at the *Ppd-B1* locus;

Sample 13 (Renan), 14 (Courtot) and 15 (Cadenza), which are represent the single haploid copy number of *Ppd-B1* gene, were used as a control for detection the 0 copy type of *Ppd-B1* gene

Supplemental **Fig. 2** Principal component analysis (PCA) of the vernalization response and photoperiod sensitivity gene alleles and the geographic origin as active variables and the two developmental phases in two years as supplemental variables in the group of 683 wheat cultivars.

Supplemental **Fig. 3** Geographic distrubtion of allele combination groups for major allele types in the *VRN-A1*, *VRN-B1*, *VRN-D1*, *PPD-D1* and *PPD-B1* genes (a) across continents, and (b) across Europe.

(a)


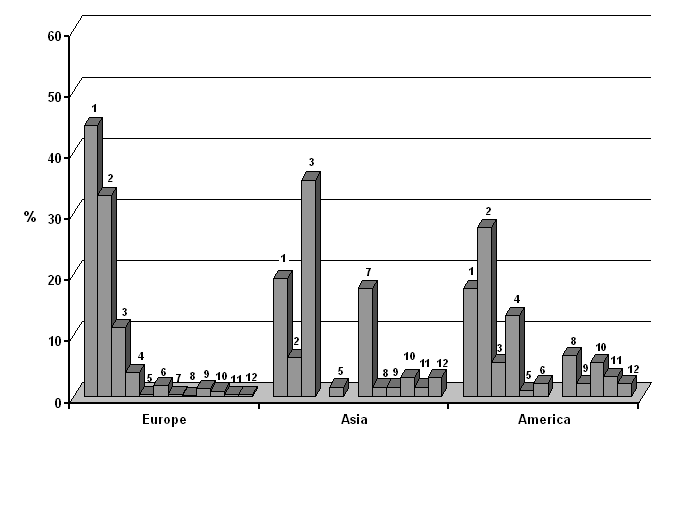


(b)


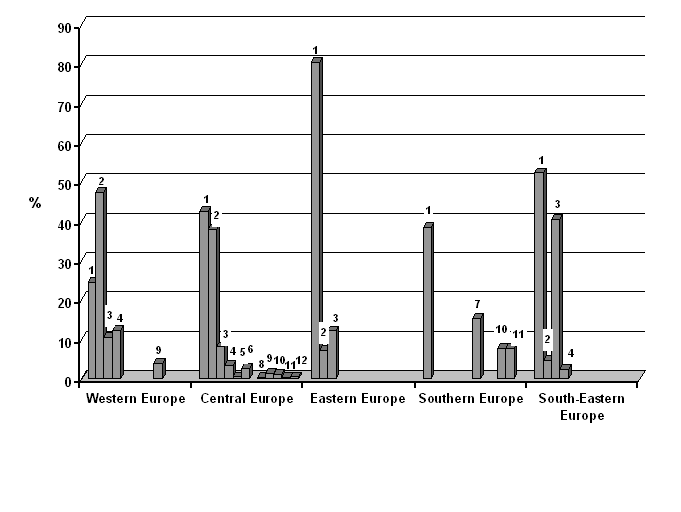


**Legend: Groups of allele combinations examined**

| **Main allele classes** | **Alleles of *VRN-A1* gene** | **Alleles of** *VRN****-B1* gene** | **Alleles of *VRN-D1* gene** | **Alleles of *PPD-B1* gene** | **Alleles of *PPD-D1* gene** |
| --- | --- | --- | --- | --- | --- |
| **1** | winter | winter | winter | sensitive | insensitive |
| **2** | winter | winter | winter | sensitive | sensitive |
| **3** | winter | winter | winter | insensitive | insensitive |
| **4** | winter | winter | winter | insensitive | sensitive |
| **5** | winter | winter | spring | sensitive | insensitive |
| **6** | winter | winter | spring | sensitive | sensitive |
| **7** | winter | winter | spring | insensitive | insensitive |
| **8** | spring | winter | winter | sensitive | insensitive |
| **9** | spring | winter | winter | sensitive | sensitive |
| **10** | winter | spring | winter | sensitive | sensitive |
| **11** | winter | spring | winter | insensitive | sensitive |
| **12** | spring | spring | winter | sensitive | sensitive |

Supplemental **Fig. 4** Correlations between the values measured in the group of 683 wheat accessions in the two consecutive years (a) for DEV49 (days from 1^st^ January to when the ears are in the upper part of flag leaf sheath), and (b) for DEV59 (days from 1^st^ January to when the ears are fully emerged).

(a)

(b)

Supplemental **Fig. 5** Average number of days required to reach the (a) DEV49 and (b) DEV59 phenophases in 12 gene allele classes of wheat cultivars based on the alleles present at the *VRN-A1*, *VRN-B1*, *VRN-D1*, *PPD-B1* and *PPD-D1* genes.
